# Double burden of malnutrition: increasing overweight and obesity and stall underweight trends among Ghanaian women

**DOI:** 10.1186/s12889-015-2033-6

**Published:** 2015-07-16

**Authors:** David Teye Doku, Subas Neupane

**Affiliations:** Department of Population and Health, University of Cape Coast, Private Mail Bag, University Post Office, Cape Coast, Ghana; School of Health Sciences FI – 33014, University of Tampere, Tampere, Finland

**Keywords:** Obesity, Overweight, Underweight, Ghanaian women, Trends, Malnutrition

## Abstract

**Background:**

Overweight and obesity are among the leading causes of mortality globally, and although previously they were mostly prevalent in developed countries, recent scanty evidence suggests that overweight and obesity in developing countries have reached high levels. Trends in overweight/obesity (BMI ≥ 25 kg/m^2^) and underweight (BMI < 18 kg/m^2^) from 1993 to 2008 and associated factors were explored among 15 to 49 years old women in Ghana.

**Methods:**

Nationally representative data were used from four Demographic and Health Surveys conducted in Ghana in 1993 (n = 4562), 1998 (n = 4843), 2003 (n = 5691) and 2008 (n = 4916). The data were analysed using logistic regression.

**Results:**

Over all, underweight increased by 28.57 % (from 10.5 %, 95 % confidence interval (CI) = 9.61–11.39 in 1993 to 7.5 %, 95 % CI = 6.76–8.24 in 2008) and 134.85 % increase in overweight and obesity (from 13.2 %, 95 % CI = 12.22–14.18 in 1993 to 31 %, 95 % CI = 29.71–32.29 in 2008) over the fifteen year period were found. Overweight was much more common in urban women (36.8 %, 95 % CI = 35.78–37.82) compared to rural women (15.6 %, 95 % CI = 14.93–16.27). Women of urban residents were more likely of being overweight (OR = 1.43, 95 % CI = 1.25–1.63) but less likely to be underweight (odds ratio (OR) = 0.33, 95 % CI = 0.30–0.36) compared to those of rural residents. Furthermore, older age, higher education, multi-parity and being rich were associated with overweight/obesity among Ghanaian women.

**Conclusion:**

Overweight and obesity are becoming a common phenomenon among Ghanaian women while underweight still remains a problem. Our study demonstrates an emerging double burden of malnutrition among Ghanaian women. Promotion of physical activity and encouraging healthy dietary habits are urgently needed to curtail obesity and overweight trends while underweight among rural women, those without higher education and those with lower wealth index can be improved through poverty reduction measures.

## Background

Overweight and obesity are regarded as one of the leading causes of mortality in the world [1] The prevalence of overweight and obesity have previously been higher in developed countries but in recent years the prevalence in many low and middle-income countries has reached the levels in developed nations [2, 3]. The rapid emergence of overweight and obesity in low and middle-income countries has been recognized as a major public health problem.

Some of the earlier studies have explored temporal trends in overweight and obesity based on nationally representative data. In Bangladesh, for example, Shafique et al. [4] collected data on 15–45 year old women with children under 5 years of age and found that overweight/obesity (BMI ≥ 25 kg/m^2^) increased among rural women from 3 to 6 % and among urban poor women from 7 to 9 % between 2000 and 2004. Balarajan and Villamor [5] investigated the trend in overweight and obesity in Bangladesh, India and Nepal and found that the prevalence of overweight/obesity had increased among ever-married 15–49 year old women from 3 to 9 % in Bangladesh, from 11 to 15 % in India and from 2 to 10 % in Nepal by 2006. However, Balarajan and Villamor [5] in the same study observed that the prevalence of underweight (BMI < 18 kg/m had decreased in Bangladesh and India (34 % in 2004 and 33 % in 2006, respectively). One of the recent studies from Nepal shows that the prevalence of overweight increased from 6 % in 2001 to 14 % in 2011 [6].

Overweight and obesity are becoming more prevalent also in many African countries as a result of urbanization, nutritional transition, adoption of western lifestyles and demographic transition [7]. Time trend analyses in an earlier study indicated that prevalence of obesity in urban West Africa has more than doubled (114 %) over 15 years [8]. Urban residents and women have particularly high risk of overweight/obesity and obesity is rising fast in women. In Ghana, the DHS surveys indicate that the percentage of women aged 15–49 overweight/obese grew from 25 to 30 % between 2003 and 2008 with the highest values among urban women [9].

Obesity is a natural consequence of over nutrition and sedentary lifestyle. However, overweight and obesity tend to be strongly associated with gender and socioeconomic status but the direction of these associations varies according to the levels of economic development [10, 11]. Contrary to high-income countries, the prevalence of overweight/obesity has frequently been highest among the wealthier, more educated and urban people in low and middle-income countries [12, 13]. Age, relative wealth, being married, having grown up in an urban environment and having parity >2 are associated with higher risk of overweight/obesity in an earlier small study in the Accra Metropolitan area in Ghana [14]. A recent review in 37 developing countries showed that obesity is occurring at a faster pace among persons of lower socioeconomic situations in countries experiencing economic development [14].

The public health consequences of the rise in obesity in West Africa are evident. However, studies examining trends in obesity and overweight and their determinants in West African countries, particularly, those using national representative sample data are scanty. Earlier studies on this subject are prevalence studies on individual countries and are often not based on representative samples, and their findings may not be applicable to the entire population. Therefore, reliable information on these trends is needed to contribute to the evidence on the menace in order to stoke up national and regional debates aimed at prevention of morbidity and associated mortality in these countries where huge disease burdens also exist in contrast to dwindling health budget. The aim of this study was to examine the trends in overweight/obesity (BMI ≥ 25 kg/m^2^) and underweight (BMI < 18 kg/m^2^) from 1993 to 2008. Additionally, we explored the associations between socio-demographic factors and overweight/obesity in 2008 among 15 to 49 years old women in Ghana. We used a high quality nationwide data, which were collected using standardised questionnaire developed for investigating demographic and health survey in several developing countries. We were also interested in studying the association between underweight and overweight and socio-demographic status among rural and urban residents.

## Methods

Data (N = 20,012) for this study were collected from the female questionnaire of the 1993 (N = 4562), 1998 (N = 4843), 2003 (N = 5691) and 2008 (N = 4916) Ghana Demographic and Health Surveys (GDHS). The Ghana Demographic Health Survey is a nationwide survey with a representative sample of women and men aged 15–49 and 15–59, respectively. All the surveys used a two stage sample based on the Ghana Population and Housing Census to produce separate estimates for key indicators for each of the ten regions in Ghana. The first stage involved selecting sample points or clusters from an updated master sampling frame constructed from the Ghana Population and Housing Census. The second stage of selection involved systematic sampling of 30 of the households listed in each cluster. This was done to ensure adequate numbers of completed individual interviews to provide estimates for key indicators with acceptable precision and to ensure a sample large enough to identify adequate numbers of under-five deaths to provide data on causes of death. The clusters were selected using systematic sampling with probability proportional to size. Each household selected for the GDHS was eligible for interview with the household questionnaire. In half of the households selected for the survey, all eligible women aged 15–49-year-old were interviewed with the women’s questionnaire. In 2008, data was not administered in one cluster due to security concerns. The data collection took place over a 3-month period, from early September to late November. The response rates were generally very high, for example, 93.8 % and 95.8 % for 2003 and 2008 respectively. The main reason for non-response was the failure to find individuals at home despite repeated visits to their household. A consent statement was read to the eligible respondent or to the parent or responsible adult for young children and unmarried women age 15–17. All who agreed to participate in the study signed a consent form either by themselves or their representatives in case of the minors and those who could not write. Ethical approval for the study protocol was given by the Ghana Health Service Ethical Review Committee in Accra, Ghana.

### Variables

Trained personnel measured height and weight using a standardized procedure. Body mass index (BMI) was the dependent variable. It was calculated by dividing body weight (kg) by height squared (m^2^). BMI was categorized into four namely; underweight-18.5 kg/m^2^, normal weight-18.5–24.9 kg/m^2^, overweight 25.0–29.9 kg/ m^2^ and obese-30.0 kg/m^2^ according to WHO recommendation (WHO, 1995).

The independent variables used in this study included place of residence (urban and rural) and age categorised as 15–19, 20–24, 25–29, 30–34, 35–39, 40–44 and 45–49 years. To increase the power for the study, age was re-categorised as 15–24, 25–34 and 35–49 years for the logistic regression analysis. The rest of the independent variables were parity (zero, one, two, three, four, five, six and seven or more), marital status (never married, currently married and formerly married), household wealth, represented by wealth index (in five categories from poorest to richest). The wealth index was constructed using data on a household’s ownership of selected assets, such as televisions and bicycles; materials used for housing construction and types of water access and sanitation facilities. The wealth index was constructed using principal components analysis. The index places individual households on a continuous scale of relative wealth. It was then categorized into five (poorest, poorer, middle, richer, and richest). Details on the construction of the wealth index and other variables can be found at http://dhsprogram.com/pubs/pdf/FR221/FR221%5B13Aug2012%5D.pdf

In addition, education (coded as; no education, primary, secondary and higher) was used. In Ghana, primary education is 6 years of schooling (from age 6 to age 12), secondary education is 6 years of schooling (from age 13 to age 18) and higher education from age 19 upwards.

### Statistical analysis

Descriptive analysis was done to show the relationship between socio-demographic status and BMI among Ghanaian women (Table 1). Next, bivariate (Table 2) and multivariate multinomial logistic regression analyses (Table 3) were conducted to assess the association between women’s socio-demographic characteristics and BMI which has three categories namely normal weight, underweight and overweight/obesity. Overweight and obesity were combined as one category to ensure enough cases for the logistic regression analysis. To examine the spatial disparities in BMI, the data were stratified into rural and urban and in the multivariate multinomial logistic regression models (Tables 3). In addition, we investigated the 15-year trend in BMI graphically and this was also done by rural and urban as well to assess disparity in the phenomenon by place of residence. Odds ratios (OR) at 95 % confidence intervals (CIs) were used to present the results of the logistic regression analyses while the trend analyses were presented using bar and line graphs. Sample weight was applied to the data to remove bias due to unequal selection probabilities. The SPSS statistical package was used to conduct data analyses.

**Table 1:** Body mass index in relation to socio-demographic characteristics among Ghanaian women aged 19–49

Indicator	BMI			
	Underweight	Normal	Over-weight	Obese
	<18.5 kg/m^2^	18.5–24.9 kg/m^2^	25.0–29.9 kg/m^2^	≥30.0 kg/m^2^
Age (in years)				
15–19	316 (13.8)	1771 (77.1)	178 (7.7)	32 (1.4)
20–24	236 (8.6)	2081 (75.4)	367 (13.3)	76 (2.8)
25–29	213 (7.6)	1907 (67.7)	524 (18.6)	174 (6.2)
30–34	151 (6.7)	1403 (62.6)	474 (21.2)	213 (9.5)
35–39	130 (6.8)	1152 (59.9)	398 (20.7)	242 (12.6)
40–44	100 (7.5)	746 (55.9)	291 (21.8)	198 (14.8)
45-49	84 (8.6)	551 (56.1)	209 (21.3)	138 (14.1)
Education				
Higher education	7 (1.9)	171 (46.8)	118 (32.3)	69 (18.9)
Secondary education	459 (7.3)	3921 (62.3)	1296 (20.6)	616 (9.8)
Primary education	336 (9.4)	2439 (68.2)	570 (15.9)	230 (6.4)
No education	428 (10.4)	3076 (74.7)	456 (11.1)	159 (3.9)
Parity				
1	195 (8.5)	1616 (70.4)	352 (15.3)	133 (5.8)
2	132 (6.2)	1394 (65.2)	437 (20.4)	176 (8.2)
3	131 (7.4)	1125 (63.2)	324 (18.2)	199 (11.2)
4	122 (8.1)	928 (61.6)	285 (18.9)	172 (11.4)
5	88 (7.9)	727 (64.9)	195 (17.4)	111 (9.9)
6	73 (8.5)	581 (67.8)	148 (17.3)	55 (6.4)
7 or more children	135 (9.7)	925 (66.7)	223 (16.1)	103 (7.4)
Marital status				
Never married	359 (11.4)	2285 (72.6)	416 (13.2)	86 (2.7)
Currently married	760 (7.6)	6542 (65.8)	1768 (17.8)	869 (8.7)
Formerly married	111 (8.8)	783 (61.8)	256 (20.2)	118 (9.3)
Wealth index				
Richest	141 (4.5)	1592 (51.2)	863 (27.7)	863 (27.7)
Richer	262 (8.6)	2023 (66.4)	517 (17.0)	517 (17.0)
Middle	263 (9.2)	2037 (71.1)	410 (14.3)	410 (14.3)
Poorer	254 (9.3)	1962 (71.7)	416 (15.2)	416 (15.2)
Poorest	305 (12.1)	1949 (77.1)	220 (8.7)	220 (8.7)
Place of residence				
Urban	347 (5.8)	3458 (57.4)	1427 (23.7)	788 (13.1)
Rural	883 (10.6)	6153 (73.8)	1013 (12.2)	286 (3.4)
Survey year				
1993	202 (10.5)	1475 (76.3)	190 (9.8)	65 (3.4)
1998	225 (9.9)	1659 (73.1)	262 (11.5)	123 (5.4)
2003	439 (8.3)	3508 (65.9)	948 (17.8)	426 (8.0)
2008	364 (7.5)	2968 (61.4)	1040 (21.5)	460 (9.5)
Total Sample	1230 (8.6)	9610 (66.9)	2440 (17.0)	1074 (7.5)

**Table 2:** The association between body mass index and socio-demographic status of Ghanaian women aged 19–49

Indicator	BMI^a,b^	
	Underweight	Over-weight/obese
	<18.5 kg/m^2^	>25.0kg/m^2^
**Age (in years)**		
15–24	1.0	1.0
25–34	0.89 (0.77–1.03)	**3.56 (3.2**–**3.95)**
35–49	**0.77 (0.67**–**0.88)**	**2.4 (2.23**–**2.74)**
**Education**		
Higher education	1.0	1.0
Secondary education	**3.00 (1.38**–**6.53)**	**0.45 (0.36**–**0.56)**
Primary education	**3.52 (1.61**–**7.71)**	**0.30 (0.24**–**0.38)**
No education	**3.56 (1.63**–**7.77)**	**0.18 (0.14**–**0.23)**
**Parity**		
1	1.0	1.0
2 or more	0.98 (0.69–1.37)	**0.52 (0.45**–**0.61)**
**Marital status**		
Never married	1.0	1.0
Currently married	**0.74 (0.64**–**0.85)**	**1.84 (1.65**–**2.04)**
Formerly married	0.90 (0.72–1.13)	**2.18 (1.86**–**2.55)**
**Wealth index**		
Richest	1.0	1.0
Richer	**1.46 (1.18**–**1.81)**	**0.43 (0.39**–**0.49)**
Middle	**1.45 (1.17**–**1.80)**	**0.32 (0.29**–**0.36)**
Poorer	**1.46 (1.18**–**1.81)**	**0.31 (0.27**–**0.35)**
Poorest	**1.76 (1.40**–**2.17)**	**0.16 (0.14**–**0.19)**
**Place of residence**		
Urban	1.0	1.0
Rural	**1.43 (1.25**–**1.63)**	**0.33 (0.30**–**0.36)**
**Survey year**		
1993	1.0	1.0
1998	0.99 (0.81–1.20)	**1.34 (1.13**–**1.60)**
2003	0.91 (0.77–1.10)	**2.27 (1.96**–**2.62)**
2008	0.89 (0.74–1.10)	**2.92 (2.52**–**3.39)**
**Total Sample**		

^a^The reference category is normal weigh

^b^Statistically significant odds ratios in bold

**Table 3:** The association between underweight and overweight, and socio-demographic status of Ghanaian women aged 19–49 in a multivariate multinomial logistic regression analysis

Indicator	Rural		Urban	
	Underweight^a,b^	Over-weight/obese^a,b^	Underweight^a,b^	Over-weight/obese^a,b^
	<18.5 kg/m^2^	>25.0kg/m^2^	<18.5 kg/m^2^	>25.0kg/m^2^
**Age (in years)**				
15–24	1.0	1.0	1.0	1.0
25–34	0.93 (0.68–1.27)	**3.25 (2.46**–**4.29)**	1.32 (0.72–2.41)	**3.64 (2.76**–**4.78)**
35–49	0.81 (0.63–1.05)	**2.21 (1.74**–**2.82)**	1.24 (0.78–1.97)	**2.57 (2.03**–**3.25)**
**Education**				
Higher education	1.0	1.0	1.0	1.0
Secondary education	2.97 (0.20–44.93)	0.52 (0.23–1.02)	1.86 (0.34–10.31)	0.84 (0.58–1.22)
Primary education	2.12 (0.14–32.22)	**0.44 (0.22**–**0.88)**	3.07 (0.54–17.25)	0.84 (0.57–1.25)
No education	2.81 (0.19–42.52)	**0.22 (0.11**–**0.44)**	3.28 (0.58–18.47)	**0.48 (0.32**–**0.72)**
**Parity**				
1	1.0	1.0	1.0	1.0
2 or more	1.1 (0.91–1.34)	**0.70 (0.58**–**0.84)**	0.98 (0.70–1.37)	**0.52 (0.44**–**0.60)**
**Marital status**				
Never married	1.0	1.0	1.0	1.0
Currently married	0.72 (0.45–1.16)	0.90 (0.56–1.46)	2.01 (0.84–4.75)	**1.48 (1.04**–**2.10)**
Formerly married	0.79 (0.47–1.32)	0.91 (0.54–1.53)	**3.51 (1.40**–**8.77)**	**1.48 (1.00**–**2.18)**
**Wealth index**				
Richest	1.0	1.0	1.0	1.0
Richer	**2.10 (1.19**–**3.70)**	**0.44 (0.34**–**0.56)**	0.98 (0.60–1.62)	**0.58 (0.49**–**0.70)**
Middle	**1.88 (1.10**–**3.31)**	**0.38 (0.30**–**0.49)**	1.33 (0.81–2.17)	**0.45 (0.36**–**0.56)**
Poorer	**2.11 (1.20**–**3.71)**	**0.41 (0.32**–**0.53)**	1.02 (0.61–1.69)	**0.44 (0.36**–**0.55)**
Poorest	**2.35 (1.34**–**4.12)**	**0.26 (0.20**–**0.34)**	1.36 (0.78–2.38)	**0.33 (0.25**–**0.45)**
**Survey year**				
1993	1.0	1.0	1.0	1.0
1998	1.05 (0.83–1.34)	**1.35 (1.04**–**1.75)**	0.60 (0.35–1.01)	**1.83 (1.42**–**2.35)**
2003	0.94 (0.73–1.20)	**1.84 (1.43**–**2.36)**	0.64 (0.41–1.00)	**1.77 (1.38**–**2.26)**
2008	0.88 (0.68–1.15)	**2.21 (1.72**–**2.84)**	**0.51 (0.31**–**0.83)**	1.03 (0.77–1.38)

^a^The reference category is normal weight

^b^Statistically significant odds ratios in bold

## Results

Table 1 shows the distribution of body mass index in relation to socio-demographic characteristics of Ghanaian women age 15–49 years. The prevalence of underweight in the latest survey (2008) was 7.5 % (95 % CI = 6.76-8.24) while 31 % (95 % CI = 29.71–32.29) of Ghanaian women were either overweight/obese. This represents 28.57 % decrease in underweight (from 10.5 %, 95 % CI = 9.61–11.39 in 1993 to 7.5 %, 95 % CI = 6.76–8.24 in 2008) and 134.85 % increase (from 13.2 %, 95 % CI = 12.22–14.18 in 1993 to 31 %, 95 % CI = 29.31–32.29 in 2008) in overweight and obesity over the fifteen year period. Consistent socio-demographic gradients were found in the prevalence of underweight, overweight and obesity.

In general, underweight was highest (13.8 %) among the youngest (15–19 years) age group of women, lowest (6.7 %) among the middle age group (30–34 years) while the oldest age group (45–49 years) had 8.6 % prevalence of underweight. On the contrary, overweight was highly prevalent (21.8 %) among the older age group (40–44 years) of women while the lowest (7.7 %) was among the youngest age group (15–19 years). Obesity was also highly prevalent (14.8 %) among women of age group 40–44 years and the least (1.4 %) was among the youngest (15–19 years) age group. Overweight and obesity were found to be positively associated with age. The prevalence of underweight increased with decreasing educational status of women with the highest prevalence among women with no education (10.4 %). Women with higher education had the highest prevalence of overweight (32.3 %) and obesity (18.9 %). It was found that the prevalence of overweight and obesity decreased with decreasing educational status.

The women with 7 children or more had the highest prevalence (9.7 %) of underweight, while the lowest (6.2 %) was found among women with 2 children. The prevalence of overweight was highest (20.4 %) among the women with 2 children and the lowest (15.3 %) among women who had only one child. Moreover, the highest prevalence of obesity (11.4 %) was found among women with 4 children while the lowest prevalence (5.8 %) was found among women with one child. Never married women had the highest prevalence of underweight (11.4 %) than currently married (7.6 %) and formerly married (8.8 %) women. The highest prevalence of overweight (20.2 %) and obesity (9.3 %) was found among formerly married women and the lowest among never married women. The prevalence of underweight was found to be highest (12.1 %) among women with the poorest quintile wealth index and decreased with increasing quintile of wealth index, while the highest (27.7 %) prevalence of overweight and obesity was found among women of the richest quintile of the wealth index and the lowest among the poorest (8.7 %). Women residing in rural area had higher prevalence (10.6 %) of underweight, whereas the women of urban residents had high prevalence of both overweight (23.7 %) and obesity (13.1 %).

The prevalence of underweight decreased from 10.5 % in 1993 to 7.5 % in the latest survey (2008). Similarly, the prevalence of overweight had increased by more than two times (9.8 % in 1993 vs. 21.5 % in 2008) and obesity (3.4 % in 1993 vs. 9.5 % in 2008) by almost three times since 1993.

Table 2 shows the association between BMI and socio-demographic status of women in bivariate analysis. Age was associated with overweight/obesity even as women of the oldest age group (35–49 years) were less likely (OR = 0.77; 95 % CI = 0.67–0.88) to be underweight. Compared to women with secondary school education or higher, women with no education were less likely to be overweight/obese (OR = 0.18; 95 % CI = 0.14–0.23) but more likely of being underweight (OR for women with no education = 3.56, 95 % CI =1.63–7.77). Similarly, parity, marital status, wealth index, place of residence and survey year were all associated with overweight/obesity. The correlation between educational attainment and wealth index was low (0.333, p < 0.001).

**Fig. 1:**
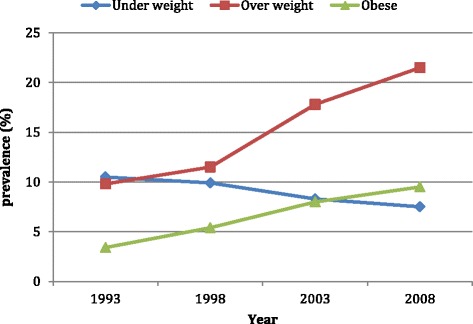
Prevalence of underweight, overweight and obesity by year of survey

**Fig. 2:**
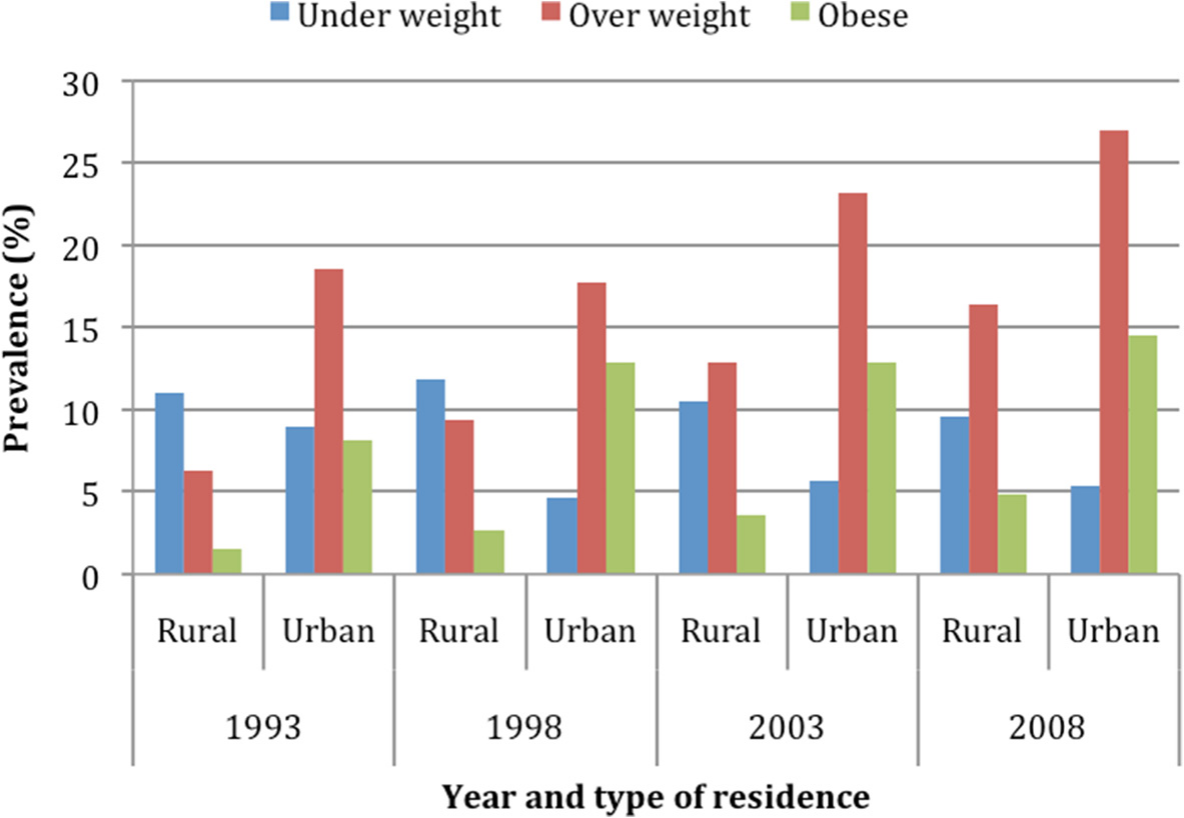
Prevalence of underweight, overweight and obesity by year of survey and type of residence

Figure 1 clearly shows that the prevalence of overweight and obesity had increased linearly in each survey. Although, not as steep as overweight/obesity, the prevalence of underweight has also decreased steadily since 1993. Figure 2 shows the prevalence of underweight, overweight and obesity among rural and urban residents in each survey. The prevalence of underweight had decreased among both rural and urban residents. However, the lowest prevalence of underweight was found among urban residents in the year 1998. Similarly, the prevalence of overweight had increased among women of both rural and urban residents. On the other hand, the increment in the prevalence of overweight and obesity was higher among women of rural residents.

The associations of underweight and overweight/obese with socio-demographic status of women among rural and urban residents from multivariate analysis are presented in Table 3. Older age was found to be associated with overweight/obesity among both rural and urban residents. However, the association was stronger among urban residents. Women with primary or no education had lesser likelihood of being overweight/obese, especially among rural residents. Women with no education were also less likely of being overweight/obese among urban residents. Women of urban residents with 2 to 5 children had higher probability of being overweight/obese compared to women who had only one child. Currently married women were found to be associated with both underweight and overweight in urban area. Poorer women of rural areas as measured by quintile of wealth index had significantly higher odds of being underweight and lower odds of being overweight/obese. Also, poorer women of urban area had lower odds of being overweight/obese. As compared to the first survey, women of rural area had significantly higher odds of being overweight/obesity in the recent surveys. However, among urban residents women had lower odds of being underweight in the latest survey and at the same time they had higher odds of being overweight/obese except in the 2008 survey year.

## Discussion

The trend and factors influencing BMI (overweight, obesity and underweight) among Ghanaian women was investigated using a large sample size data collected nation-wide over a 15-year period. This is the first study to report trends in the prevalence of overweight and underweight among women in Ghana using nationally representative sample. Disparities in overweight, obesity and underweight were found by age, level of education, parity, marital status, wealth index and survey year. Furthermore, there were rural–urban differences in both underweight and overweight. In the general Ghanaian women population, the prevalence of overweight and obesity has consistently increased over the 15-year period (1993–2008). However, the prevalence of underweight was nearly the same between 1993 and 1998, and slightly decreased thereafter. By rural–urban stratification, it was observed that the prevalence of overweight and obesity have consistently increased from 1993 to 2008 in urban settings. The only exception was that in 1998 the prevalence of overweight in urban areas decreased slightly.

Obesity is a well-known risk factor for several chronic illnesses including type II diabetes, hypertension and scores of cardiovascular diseases. Underweight on the other hand, particularly, among women have direct effect on their health e.g. anaemia and indirectly contributes to maternal and infant mortality. The relative high prevalence of overweight and obesity found among Ghanaian women and the increasing trends observed warrant public health attention. Ghana and many African countries are battling with many existing infectious diseases including malaria, tuberculosis and HIV and AIDS.

Socio-demographic differences in underweight and overweight/obesity by level of education, parity, marital status, wealth index were similar to those found in the Accra Metropolitan area [14]. The increasing prevalence in underweight and overweight/obesity may be explained by the changing nutritional and lifestyle patterns in Ghana. Many African countries have experienced rapid economic growth and development over the past one and half decades. This rapid growth has led to urban lifestyles including changes in food consumption pattern such as consumption of refined food due to the globalisation of the food market [10, 11]. Economic growth, modernisation and globalisation may have also contributed to involvement in more sedentary lifestyles such as motorised lifestyle in Ghana, particularly, in the urban areas. With respect to the association found between wealth index and obesity and overweight, one plausible explanation could be the cultural perception in Ghana, and in Africa in general, which favour large body size [15].

Although in Western countries, overweight is more prevalent among the less educated and those at the lower end on the socioeconomic ladder, in developing countries, a different picture emerged from the literature. Education is positively related to overweight and obesity and the higher a person’s socioeconomic status, the higher the probability of being overweight or obese [12, 13]. The present study confirms this relationship found in developing countries.

Apart from economic growth and modernisation, the contextual meaning of body size might be playing a role in the increasing prevalence of overweight and obesity. In Ghana, cultural values favour large body size [15]. Being fat is often misconstrued as a sign of wealthy living. What is intriguing is the fact that whereas overweight and obesity are increasing, underweight generally remained the same over the period, and among rural women, there has not been any significant change in underweight. This study reveals a double burden of malnutrition that has been reported in other developing countries [5, 6]. With regards to the rural–urban differences, our finding is similar to a recent study from Nepal, which also reported marked differences in the phenomenon among women by rural–urban setting [6]. Several plausible explanations could account for the rural–urban disparities reported here. In the rural areas women mostly engage in agricultural and other activities, which are physical and therefore unlikely to gain as much weight as the urban women. Moreover, women in rural areas are less exposed to western lifestyle and the nutritional transition in the urban areas is not likely to be prevalent in the rural setting. Furthermore, it seems that whereas urban women have more than enough to eat, women in rural have less.

Our study has some strength. Firstly, the data were based on large nationally representative surveys conducted at four time points and the response rate was very good (93 to 96 %) among the eligible women in all four surveys. Therefore, the results are considerably generalizable to the whole country. Secondly, data on the body weight and height were measured by trained study personnel with similar measurement equipment making the data comparable. Thirdly, the surveys used standardized methods comparable to multiple countries. There are also some limitations that are worth discussing. We did not have data on waist circumference which would have allowed examination of trends in abdominal obesity. Additionally, no data were available on behavioural or other factors that could have explained the observed changes in the prevalence of overweight among women.

## Conclusion

In conclusion, double burden of malnutrition, comprising overweight/obesity and underweight exists among Ghanaian women. Moreover, overweight and obesity have continuously risen at the same time underweight did not significantly reduce during the 15-years period. Older age, higher education, parity, rich quintile in wealth index and urban residents were associated with overweight/obesity problems among Ghanaian women. These finding suggest that unless malnutrition is addressed, in the future there will be inequalities in overweight and underweight related morbidity and mortality. On one hand, national level strategies of promoting healthy lifestyle to prevent further increase in overweight and obesity and consequently in the incidence of the related chronic diseases should urgently be developed and implemented. On the other hand, strategies are needed to prevent underweight among Ghanaian women.

### Ethical approval

No ethical approval was required for this particular analysis since ethical clearance was already obtained from the Ghana Health Service for the conduct of the Ghana Demographic and Health Survey.
